# Out of synchrony with the imagined self: A scoping review of self‐concept in premature ovarian insufficiency and early menopause

**DOI:** 10.1111/bjhp.70104

**Published:** 2026-08-20

**Authors:** Lisa M. Seberry, Subhadra Evans, Antonina Mikocka‐Walus, Leesa M. Van Niekerk

**Affiliations:** ^1^ University of Tasmania College of Health, School of Psychological Sciences Hobart Tasmania Australia; ^2^ School of Psychology Deakin University Burwood Victoria Australia

**Keywords:** early menopause, healthcare, premature ovarian insufficiency, self‐concept

## Abstract

**Objective:**

Premature ovarian insufficiency (POI) and early menopause (EM) can have life‐altering physical and psychological impacts, with potential consequences for an individual's sense of self. However, understanding of these potential consequences is limited, with much of the literature generalizing such experiences from naturally occurring menopause. This scoping review aimed to increase understanding around the alteration of self‐concept in adults, presumed female at birth, diagnosed with POI or EM.

**Methods:**

In line with the Joanna Briggs Institute (JBI) protocol for scoping reviews, database searches were conducted, with eligible studies consisting of peer‐reviewed literature exploring how self‐concept and/or its associated constructs are impacted by POI or EM.

**Results:**

Database searches identified 473 manuscripts, with 35 full‐text manuscripts reviewed and 12 deemed to be eligible for inclusion. Impacts to physical, social and emotional self‐concept were indicated across studies, and detailed via content analysis. Contextual factors were suggested to greatly influence the extent of self‐concept impact. The impact of spontaneous versus medically induced POI and/or EM on self‐concept showed some variation; however, detailed explanation for this was limited across the manuscripts.

**Conclusion:**

This review provides greater understanding of the disruptive nature of POI or EM, potentially impacting all aspects of self‐concept—physical, social and emotional. Such outcomes emphasize the importance of recognizing the unique experiences of POI and EM and the need for individualized understanding and care. Further research is needed to better understand the experiences of these individuals and their support and treatment needs.

**Statement of Contribution**

**What is already known**

- Premature ovarian insufficiency (POI) and early menopause (EM) are associated with significant physical and psychological impacts.
- Much of the understanding of these impacts is generalized from naturally occurring menopause.
- POI and EM are associated with changes to self‐concept.

**What does this study add**

- A narrative synthesis of existing published mixed methods evidence of the impacts to self‐concept associated with POI and EM.
- An understanding of the themes and experiences associated with physical, emotional and social self‐concept in women with POI and EM.
- An understanding of the role of context factors in influencing self‐concept challenges for women with POI and EM.

## INTRODUCTION

Premature ovarian insufficiency (POI) and early menopause (EM) are life‐changing diagnoses, yet those diagnosed repeatedly report feeling ignored and misunderstood (Singer, 2019). Experiences from natural menopause have been generalized to POI and EM, although such generalizations undermine the unique experiences of POI and EM (Loxton et al., 2021; Singer, 2019). Natural menopause, a normative age‐related biological transition typically occurs midlife (age 45–54 years), whereas POI and EM involve the premature loss of ovarian function. POI occurs before 40 years of age, and EM occurs between 40 and 45 years of age, can be spontaneous or medically induced/iatrogenic, and are defined by loss of ovarian activity, low estradiol levels, and elevated gonadotropins (Hamoda & Sharma, 2024; Webber et al., 2016). In EM, these symptoms denote a permanent cessation of ovarian function, yet while reproduction is unpredictable with POI, it is not impossible (Hamoda & Sharma, 2024; Singer, 2019). These distinctions have only recently been acknowledged within the literature, with much of the research conflating the two diagnoses (Singer, 2019). With consideration to aetiology, POI or EM should not be understood through age and fertility changes alone, but rather as multifactorial diagnoses, with life‐altering physical and psychological impacts (Faubion et al., 2015; Singer, 2019).

The physical menopausal symptoms associated with POI and EM include hot flushes/flashes, mood changes, vaginal dryness, night sweats, sleep disturbance, low libido and dyspareunia (painful sexual intercourse), as well as more long‐term health problems in relation to bone health, cardiovascular health and sexual health (Hammond & Marczak, 2025; Li et al., 2020). While these impacts are experienced in natural menopause, they are often more severe in POI and EM (Hammond & Marczak, 2025). The deficiency of oestrogen and other associated hormones and hormonal mechanisms prior to the median age of menopause increases risk of early morbidity and mortality, with oestrogen treatment mitigating only some consequences (Ossewaarde et al., 2005; Shuster et al., 2010). The psychological impacts of POI and EM are influenced by physiological changes, the diagnostic journey and the social and cultural environment in which the diagnoses are experienced (McDonald et al., 2022; Pal & Santoro, 2002). The rapid decline in hormones such as oestrogen and subsequent physical symptoms and changes influence mood disturbances and cognitive changes (Li et al., 2020; Singer, 2019). Ongoing psychological distress persists with symptoms of depression, anxiety and somatization, as well as reduced life satisfaction and self‐esteem (Guerrieri et al., 2014; Liao et al., 2000; Schmidt et al., 2006). Feelings of uncertainty, shock, grief, sadness and anger often fluctuate as individuals try to understand and manage their diagnosis (McDonald et al., 2022).

These experiences are interwoven with the personal meaning gathered from diagnosis, which is influenced by the social and cultural context (Singer, 2019). With this, interpersonal relationships are not only impacted by a POI or EM diagnosis but also have a role in shaping how one conceptualizes and experiences the diagnosis (Johnston‐Ataata et al., 2020). Notably, changes in fertility and/or sexual intimacy may differentially impact romantic relationships depending on factors such as, stage of relationship, identification with gender and age norms and individual and shared goals (Johnston‐Ataata et al., 2020). For instance, a lack of ovarian activity may lead to feelings of isolation from the self and peers, especially if an individual perceives this as an important part of their femininity and/or sense of womanhood (Johnston‐Ataata et al., 2020; Singer & Hunter, 1999; Singer 2019). Accordingly, for many with POI or EM, aspects of self‐concept may be fractured and reimagined (Hammond & Marczak, 2025).

Self‐concept may be viewed through a multidimensional hierarchical model in which the individual domains of self‐concept (i.e., physical, emotional, social) interconnect to form a global self‐concept (Mercer, 2012; Shavelson et al., 1976). Physical self‐concept encompasses how one perceives their body in terms of appearance, functionality and health. Emotional self‐concept reflects one's perception of their internal emotional world, their own competencies and/or abilities relating to emotional experiences (i.e., emotional stability). Social self‐concept indicates how one perceives themselves in social contexts in relation to others including perceived roles, shared values and belonging to certain groups (Mercer, 2012). Constructs such as self‐esteem, self‐confidence, self‐image, body image and identity are closely related to self‐concept, but serve different psychological functions (Bailey, 2003; Mercer, 2012). Self‐esteem and self‐confidence are linked to the evaluative aspects of self‐concept, with self‐esteem reflecting an individual's overall understanding of their worth or value, and self‐confidence referring to an individual's belief about their capabilities and skills (Harter, 1999). Body image encompasses the evaluations made in relation to our physical self, predominantly linking into the physical dimension of self‐concept (Bailey, 2003). Identity, while very interrelated with self‐concept, focuses on an individual's sense of self derived from a specific social context and/or community, rather than a broader domain (i.e., emotional self‐concept; Mercer, 2012).

Common experiences across the lifespan (i.e., increased independence, relationship formation, children, career), as well as disruptions to such experiences (i.e., chronic illness) can impact self‐concept, shifting the content and nature of self‐descriptions (Charmaz, 2002; Mazalin et al., 2024; Mercer, 2012). Accordingly, research suggests that disruptions experienced with POI and EM impact physical, emotional and social self‐concept (Hammond & Marczak, 2025; Singer, 2019). Many individuals with POI or EM identify a significant shift in how they conceptualize their health, perceiving a shift from being healthy individuals to an ill person who is damaged or broken (Singer, 2019). For some, their young adult identity may be challenged due to a perceived incongruence between their chronological and biological age (Boughton, 2002; Moukhah et al., 2021). Yet others feel that their diagnosis threatens their femininity, with changes in their body's function and appearance influencing feelings of alienation from their past selves (Golezar et al., 2020; Orshan et al., 2001; Singer, 2019).

Research on self‐concept in POI and EM shows considerable variability in how the construct is defined and measured. While some studies explicitly examine self‐concept, others focus on related constructs such as self‐esteem, body image and identity without directly naming self‐concept (Hammond & Marczak, 2025; Johnston‐Ataata et al., 2020; Moukhah et al., 2021). This inconsistency is further compounded by the predominance of qualitative research, where these terms are often used interchangeably, limiting comparability across studies. Although existing systematic reviews have examined broader psychosocial outcomes and quality of life in POI (Li et al., 2020; McDonald et al., 2022), they have not specifically addressed self‐concept, its associated constructs or included EM populations. Consequently, a gap remains in understanding how these diagnoses uniquely affect self‐concept. Addressing this gap is important for informing targeted psychosocial interventions that move beyond generalizations from natural menopause and instead respond to condition‐specific changes in self‐concept (Hammond & Marczak, 2025; Singer, 2019). Therefore, this study aims to conduct a scoping review of self‐concept and related constructs in adults presumed female at birth diagnosed with POI or EM.

## METHODS

A scoping review was conducted in line with the Joanna Briggs Institute (JBI) protocol for scoping reviews (Peters et al., 2020). The review was registered with the Open Science Framework on 14 January 2025 (https://osf.io/mce56/). Table 1 details the information sources, inclusion and exclusion criteria and associated search terms for the scoping review.

**TABLE 1:** Information sources and scoping review inclusion criteria.

	Inclusion criteria	Exclusion criteria	Search terms
Setting	All geographic locations; Clincs; Research facilities; Community setting	Not applicable	Not applicable
Population	Human participants; Women and transgender or gender diverse (TGD) individuals; Presumed female at birth; Adults aged 18 years or older; POI or EM diagnosis; Spontaneous or medically induced POI and EM; All cultural backgrounds and ethnicities	Individuals presumed male at birth; Adolescent samples; Childhood samples; Samples with known genetic or chromosomal causes, such as fragile X syndrome or Turner Syndrome	“primary ovarian insufficiency” OR “early menopause” OR “primary ovarian insufficiency” OR “premature menopause” OR “premature ovarian failure”
Construct	Explicit use of the term self‐concept and/or associated constructs (e.g., self‐esteem, identity, body image)	No discussion of self‐concept and/or associated constructs.	“self‐concept” OR “self‐identity” OR “sense‐of‐self” OR “self‐image” OR “self‐worth” OR “self‐competence” OR “self‐belief” OR “self‐descriptions” OR “self‐confidence” OR “self‐esteem” OR “self‐perception” OR “social‐identity” OR “self‐view” OR “self‐value” OR “body‐image” OR “self‐appraisal” OR “self‐assessment” OR “self‐evaluation” OR “self‐rating”
Information sources	Peer reviewed studies; Written in or translated into English; Qualitative, quantitative, and mixed methodology studies (trials, case studies, observational studies, cross‐sectional, longitudinal, descriptive); Grey literature; There were no restrictions for year of publication	Conference abstracts; Systematic reviews; Scoping reviews; Book chapters; Dissertations; Posters	Not applicable

### Search strategy

The preliminary search strategy was formulated by the research team, with subsequent iterative consultation with a research librarian from the University of Tasmania to support selection of refined search terms. A three‐step search strategy was utilized. First, a search of two online databases (Scopus, Medline) aided an initial identification of articles relating to POI, EM and self‐concept. Second, titles and abstracts of relevant studies provided relevant text words. Index terms/subject headings were identified through article descriptions, and MeSH terms were identified via the PubMed MeSH (Medical Subject Headings) database. The search terms were applied to four scientific databases between November 2024 and January 2025: Scopus, Medline, Web of Science and PsycINFO, with no date restrictions applied. Third, reference lists of full‐text articles were screened for additional studies that may have been overlooked in the initial online search.

### Study selection and data extraction

Following search completion and removal of duplications, two researchers independently screened all titles and abstracts and full texts via Covidence Systematic Review Software (Veritas Health Innovation, 2021, Melbourne, Australia, 2024; available at www.covidence.org). The researchers were blinded to each person's inclusion and exclusion decisions. Discrepancies were automatically identified by Covidence and were resolved via verbal discussion and consensus, with reference to the predefined eligibility criteria. In line with PRISMA‐ScR guidelines, the study characteristics and findings are presented in table format. A narrative synthesis was undertaken to answer the scoping review questions. As both qualitative and quantitative studies were eligible for inclusion, a convergent integrated approach was used. All findings were synthesized using content analysis, with data grouped into categories based on conceptual similarity rather than study design (Peters et al., 2020). Quantitative results were summarized descriptively and integrated with qualitative findings within a shared thematic framework. Regular team discussions were conducted to refine categories and reach consensus on the final themes.

## RESULTS

**FIGURE 1:**
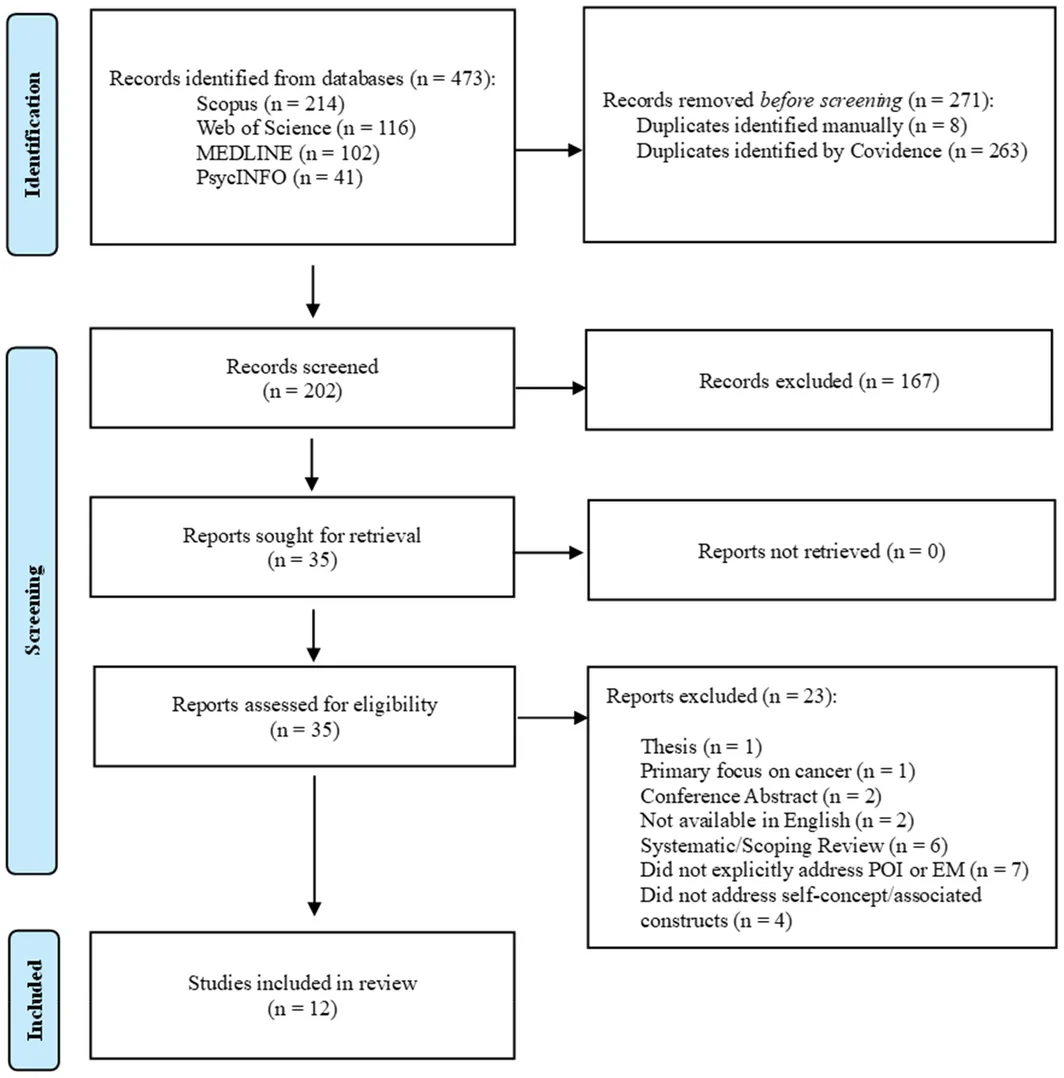
PRIMA‐ScR flowchart of study selection (Page et al., 2021).

Database searches identified 473 manuscripts. After 271 duplicates were removed, 202 studies remained for screening. See Figure 1 for the PRISMA‐ScR flow chart, indicating the study selection process. The characteristics of the 12 included studies are outlined in Table 2. The studies included exclusively female participants, with age at time of study ranging between 19 to 65 years, with most studies predominantly including participants from white western backgrounds (i.e., Australian, British, Canadian, Irish), with minor inclusion of other backgrounds (i.e., Asian, Hispanic, Māori, Italian). One study focused solely on Iranian women and another on Korean women.

**TABLE 2:** Overview of general study characteristics.

Author, year	Location, study design	Aims	Characteristics of participants	Menopause history	Relationship status, children, HRT	Self‐concept and related constructs^a^
Boughton (2002)	Australia, qualitative study	To understand the experience of menopause for women who were not yet 40 years old	Females (*n* = 35), heterosexual, white Anglo‐Saxon & Celtic background, age at study not reported	POI, spontaneous (*n =* 22), medically induced (*n =* 13), age at menopause ranged from early 20s to 30s	23 were married at time of study, mean number of children was 1.3, 8 had no children, 27 had at least one child. Information pertaining to HRT was not reported	Self‐identity, sense of self, self‐view, body image, social identity, *self‐concept, self‐confidence, physical self‐concept*
Boughton and Halliday (2008)	Australia, qualitative study	To report young Australian women's lived experiences of premature menopause and their reflection upon gaining a diagnosis	Females (*n* = 35), heterosexual, predominantly white Anglo‐Saxon & Celtic, one Asian participant, age at study not reported	POI, spontaneous or medically induced not indicated, age at menopause ranged from 19 to 39 years	Information pertaining to relationships, children, or HRT was not reported	Sense of self, *self‐view*, *self‐concept self‐identity*
Deeks et al. (2011)	Australia, cross‐sectional study	To understand the experience of diagnosis, sources of information, and perceptions of effective treatment and to examine psychosocial impacts (i.e., depression, anxiety, body image)	Females (*n* = 77), mean age of 34.8, sexuality and cultural & ethnic background not reported	POI (*n =* 25) spontaneous (*n =* 29), medically induced (*n =* 23), premenopausal controls, average time since diagnosis was 3.8 years, age at menopause or diagnosis not reported	Mean number of children for women with POI ranged from 0.8 to 1.1. Information pertaining to relationships or the number of women using HRT was not reported	Body image
Golezar et al. (2020)	Iran, qualitative study	To explore the factors affecting the quality of life of women with POI	Females (*n* = 16), mean age of 36.7, Iranian nationality and Farsi‐speaking, sexuality not reported	POI, spontaneous, age at menopause or diagnosis not reported	10 were married, 2 were divorced, 4 were single, 6 had at least one child, 10 had no children. The number of women using HRT was not reported	Self‐concept, self‐image, body image, self‐confidence, self‐identity, *social identity*
Gosset et al. (2023)	France, cross‐sectional study	To evaluate the impact of vaginal symptoms of genitourinary syndrome of menopause on the sexual functioning and quality of life of women with idiopathic POI	Females (*n* = 88), mean age of 41.6, sexuality or cultural & ethnic background not reported	POI, spontaneous. Mean age at diagnosis was 32.6 years, average time since diagnosis was 9 years	55 were in a relationship, 51 were taking HRT. Information pertaining to children was not reported	Self‐concept, body image
Johnston‐Ataata et al. (2020)	Australia, qualitative study	To examine the enmeshment of personal relationships with the formation of early menopausal subjectivities	Females (*n* = 25), aged between 28 and 51 years, 19 heterosexual, 2 bisexual, 3 lesbian, 1 not stated, 18 were Australian, British, Canadian or Irish, 1 Chinese, 1 Greek‐Australia, 1 Italian, Portuguese, German, 1 Māori, 1 ‘Northern European’, 2 not stated	15 POI & 10 EM, 10 spontaneous & 15 medically induced, age at diagnosis ranged between 25 to 44 years	17 were in a relationship, 8 were single, 12 had at least one child, 2 were pregnant, 11 had no children. The number of women using HRT was not reported	Self‐identity, sense of self, self‐confidence, self‐concept, *social identity, sexual identity*
Liao et al. (2000)	United Kingdom, cross‐sectional study	To investigate the psychological well‐being of women who had experienced menopause before the age of 40 years	Females (*n* = 64), mean age of 30.8 years, majority white British (*n* = 56), sexuality was not reported on	POI, spontaneous, mean age of diagnosis was 24.6 years, mean time since diagnosis was 6.4 years	32 were married, 14 in relationship but not married, 18 were single, 18 had at least one child, 46 had no children, 52 wanted to have children in the future, 54 were taking HRT	Self‐esteem, body image, *sexual identity*
Moadel et al. (1995)	United States, cross‐sectional study	To examine the psychosexual adjustment of women who experience premature menopause due to cancer therapy and to assess the severity of menopausal symptoms, relationship adjustment, body image, and psychological distress	Females (*n* = 34 subject sample, *n* = 24 comparison group)^b^, mean age of subject sample was 36 years, 32 white, 1 Hispanic, 1 other	POI, 34 medically induced, 24 premenopausal, mean age of POI diagnosis was 27 years, mean time since diagnosis was 6.5 years	24 were married, 10 were in a relationship, 11 had at least one child, 23 had no children, 34 were taking HRT	Body image
Ryu et al. (2022)	Korea, cross‐sectional study	To investigate the association between age at menopause and select depressive behaviours among middle‐aged Korean women	Females (*n* = 2, 232), aged between 40 and 65 years, Korean, sexuality was not reported on	25 POI, 114 EM, & 2093 NM, spontaneous, specific age & age range at menopause or diagnosis not reported	96% with POI, 97.4% with EM, and 98.4% with NM were married, 92% with POI, 93% with EM, and 97% with NM had at least one child. Information pertaining to HRT was not reported	Self‐esteem
Singer (2012)	United Kingdom, cross‐sectional mixed methods study	To investigate women with premature menopause and their experience of diagnosis, perception of cause, treatment received, main concerns, perceived long‐term consequences and impact on psychological well‐being	Females (*n* = 136), aged between 19 and 61 years, majority white British (85%), sexuality was not reported on	POI & EM^c^, spontaneous & medically induced, mean age of diagnosis was 31 years, time since diagnosis was within the previous 6 months for 41% and between 6 months and 3 years for 30%	71% were married and/or were in a relationship, 63% had no children, 69% were taking HRT	Self‐identity, sense of self, self‐worth, self‐esteem, self‐confidence, *self‐concept*, *sexual identity*
Singer and Hunter (1999)	United Kingdom, Qualitative	To explore how women with early menopause describe their experience and to examine how these narratives relate to social discourse of reproduction, gender and age. A second aim was to consider how women maintain a sense of self and identity in the face of potential negative discursive practices relating to menopause	Females (*n* = 13), mean age of 31 years, predominantly white and one Asian participant, sexuality was not reported on	POI & EM^c^, spontaneous & medically induced, mean age of menopause was 28 years, mean age of diagnosis was 29 years	7 were married and/or in a relationship, 6 were single, 8 had children, 5 had no children, 11 were taking HRT and/or other forms of medication	Self‐identity, sense of self, self‐image, self‐worth, self‐esteem, body image, *self‐concept*, *social identity*, *sexual identity*
Singer et al. (2011)	United Kingdom, cross‐sectional mixed methods study	To investigate women's experiences of diagnosis, perception of cause, treatment, their main concerns, the long‐term consequences and the impact on self‐esteem, sexual functioning and health‐related quality of life	Females (*n* = 136), mean age of 38.7 years, 116 white British, 8 Asian, 5 Black, 6 other, sexuality was not reported on	POI and EM^c^, spontaneous and medically induced, mean age of diagnosis was 31 years, mean time since diagnosis was 8 years	90 were married, 58 had at least one child, 9 were taking HRT	Self‐identity, self‐image, self‐esteem, body image, self‐confidence

*Note*: Study design terminology was standardized; ‘cross‐sectional’ and ‘cross‐sectional observational’ were considered equivalent.

Abbreviation: NM, natural menopause.

^a^ Related constructs inferred/alluded to in the study are presented in italics.

^b^ Sample information refers to subject sample, unless indicated otherwise.

^c^ Most POI, with premature menopause and early menopause previously defined as 40 years and under.

Studies focused on women with either POI (*n* = 7) or both POI and EM (*n* = 5). Most studies included both spontaneous and medically induced (*n* = 7) or solely spontaneous (*n* = 4), while one study included just medically induced menopause. For studies focusing on POI, age of menopause or diagnosis ranged from 19 to 39 years, and the average time since diagnosis was 6.4 years (*n* = 4). The mean age of diagnosis was 30 years (*n* = 3) for studies that included both POI and EM. Due to changes in terminology and/or definition of POI and EM over time, some studies appeared to focus on those with POI yet include individuals aged 40; therefore while such studies include those with POI and EM, the predominant sample was POI.

### Impact of POI and EM on self‐concept and associated constructs

Self‐concept was variably reported and most prominently inferred to via associated constructs. The most frequently reported construct across studies was body image, followed by self‐identity/identity, self‐confidence, self‐esteem, sense of self, self‐image, self‐worth, self‐concept, self‐view and social identity. Some constructs which were less frequently addressed were often inferred, such as self‐concept and social identity. Sexual identity was only inferred in studies (*n* = 4) rather than explicitly discussed. The qualitative and mixed methods studies explored a wider range of self‐concept related constructs, while the quantitative focus was narrower, most commonly investigating impacts of POI and/or EM on body image and self‐esteem. Table 3 broadly summarizes the impacts of POI and EM on self‐concept and associated constructs reported in each study.

**TABLE 3:** Impacts of POI and EM on self‐concept and associated constructs.

Author, year	Findings on the impacts of POI and/or EM on self‐concept and related constructs
Boughton (2002)	POI led to various disruptions to the women's lives and/or self in relation to their ‘biological body’ experience versus their ‘lived body’
Boughton and Halliday (2008)	The experience of looking for a diagnosis caused significant disruption to the women's lives, contributing to feelings of uncertainty around their body and experiences. Stereotypes around menopause, reinforced by health care professionals and the women themselves, played a significant role in the women's interpretation of their experiences
Deeks et al. (2011)	Impacts of POI on self‐concept associated constructs, with some differences depending on POI cause (i.e., spontaneous, surgical, chemical)
Golezar et al. (2020)	POI can distort self‐concept due to factors such as disease stigma and threatened identity
Gosset et al. (2023)	Vaginal symptoms and/or GSM associated with POI can have a significant impact on self‐concept and body image
Johnston‐Ataata et al. (2020)	The extent to which EM or POI influenced sense of self and self‐identity was influenced by various interlinked factors relating to the women (‘location’ in imagined biographies, cause, identification with norms related to age and gender, symptoms, concerns regarding physical changes, support, opinions of family and friends)
Liao et al. (2000)	POI is associated with higher psychological distress than the general population, with impacts to some areas of self‐concept. Authors suggest that such impacts are influenced by age at diagnosis, previous psychological difficulties, relationships, and children
Moadel et al. (1995)	The impact to self‐concept associated constructs (i.e., body‐image) may be influenced by the experience of cancer and the connected meaning‐making (i.e., focus on survivorship rather than loss)
Ryu et al. (2022)	Those with POI and EM are more likely to experience negative impacts to self‐esteem than those experiencing menopause after 45 years, with greater impact for those experiencing menopause earlier
Singer (2012)	Impact of POI showed some variation depending on individual factors such as cause, age, and experience with health care professionals, however most experienced a significant sense of loss with impacts to various aspects of self‐concept and associated constructs. This was especially evident in the initial stages of symptoms and diagnosis
Singer and Hunter (1999)	Impact of POI on self‐concept changed overtime, with more extreme negative experiences initially, which shifted over time to include some positive or neutral experiences, feelings with efforts to reimagine the self. Social discourses of reproduction, gender, and ageing associated with the early menopausal women had implications for the women's self‐concept
Singer et al. (2011)	Women had wide ranging concerns arising from POI, such as, fertility, levels of stress, relationships, and experiences with health care professionals, which impacted on self‐concept and associated constructs

**FIGURE 2:**
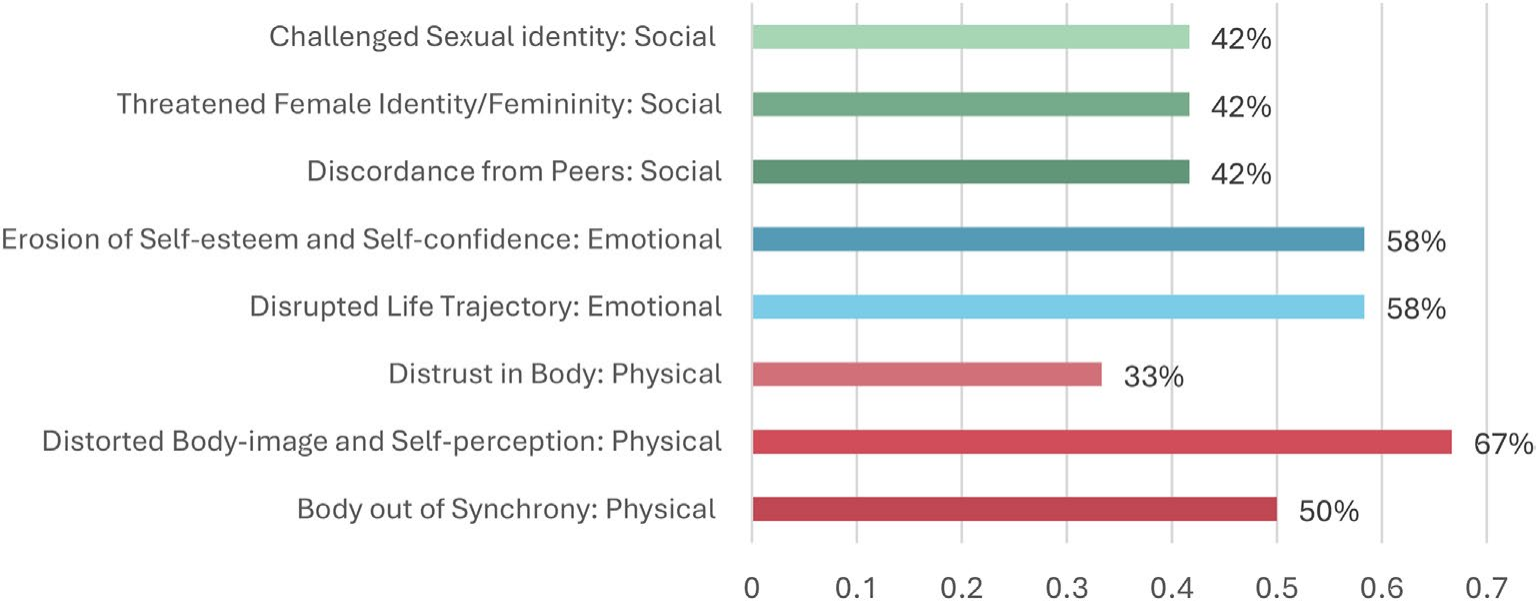
Percentage of studies identifying each self‐concept category.

All studies highlighted the potentially disruptive nature of POI and/or EM on the women's lives, many detailing the sense of uncertainty and the questioning of the self often associated with diagnosis (Boughton & Halliday, 2008, p. 569 ‘The uncertainty of symptoms and not being given an explanation or diagnosis led some women to a point of feeling everything might have been in their heads or that they were “going insane”’). Impacts to physical, social and emotional self‐concept were explored across studies, with review of such impacts, via content analysis, indicating eight distinct categories. Some categories could fit into more than one domain of self‐concept, reflecting the interconnected nature of aspects of self‐concept, however, each were assigned to the area of ‘best‐fit’ to aid presentation of results. Figure 2 provides a graphical representation of the percentage of studies which described each category.

### Physical self‐concept

Impacts to physical self‐concept were most frequently reported on across the reviewed studies, making up 38% of all category references (see Figure 2). Distorted body‐image and self‐perception were described by 67% of studies (*n* = 8), representing the most prevalent impact of POI and EM on self‐concept reported. This category reflected the women's shifted perception of their physical appearance and abilities due to menopause, often relating to fears that they were ageing prematurely and were less physically attractive and youthful compared to others their age. Such fears often aligned with negative stereotypes relating to menopause. The image associated with POI or EM often led the women to perceive themselves as less physically or sexually attractive and rather prematurely aged and withered. Increased self‐surveillance of the body often followed as an attempt to regain control over the perceived rapid changes. The experience of the women in Boughton's (2002) study was like that of other studies:

‘The women in this study lost their sense of their outward body appearance because they had been exposed to the socially contrived view of the menopausal woman, always an older woman, “the grandmotherly type”’ (Boughton, 2002, p. 427).

Half of the studies (*n* = 6) described body out of synchrony as an impact of POI and EM on self‐concept. Body out of synchrony reflected some women's experience of feeling as though their physical body was out of synchrony from their age and usual abilities. Many women acknowledged knowing they were young in relation to their age, yet their body felt old, with a sense of being caught between two polarities (Singer & Hunter, 1999, p. 73: ‘*being young (“me”) and menopausal (“not me”)*’; Boughton, 2002, p. 427: ‘*menopause meant that biologically their body was ahead of their embodied self – it was out of synchrony*’). This experience reflected a self‐body disruption and often fostered a sense of disconnect from their body because of POI or EM.

Distrust in body was described by one third of studies (33%, *n* = 4), encompassing the experience of uncertainty and ambiguity around the women's bodily experiences (Boughton & Halliday, 2008, p. 568): ‘*the vagueness led to women feeling they could not interpret symptoms with certainty*’. As women experienced menopause earlier than the average or expected age, many viewed their body as problematic, feeling as though it was malfunctioning or at odds with itself. This was perpetuated by responses from HCPs, family and friends, adding to a pervasive sense of uncertainty and doubt. Since the image of the menopausal woman did not align with the women diagnosed, they were often led to feel that their bodies couldn't be trusted and rather were ‘*a site of sickness needing treatment*’ (Singer & Hunter, 1999). Boughton and Halliday (2008) captured this theme in their findings:

‘With many women being told they were just too young for menopause, they were left with a sense that they were “going insane”, that it must have been “all in their heads”…The uncertainty of what was happening to them was a significant factor in their feelings of insanity.’ (p. 570–571).

### Social self‐concept

Impacts to social self‐concept made up just under one third of all category references (*n* = 32%). Discordance from peers was described in 42% of studies (*n* = 5). This category described a perceived separation from people their age, with the women feeling that they could not relate to their peers, nor entirely identify with those experiencing natural menopause. This often led to feelings of isolation, with a sense of confusion around where they belong, with some women feeling a sense of inferiority to their peers. The following words were often used by women to describe themselves and their perceived otherness: ‘*abnormal*’, ‘*not natural*’, ‘*deficient*’, ‘*useless*’, ‘*different*’, ‘*outsider*’ and ‘*freak*’ (Golezar et al., 2020; Singer, 2012; Singer & Hunter, 1999). The women in Singer and Hunter's study (Singer & Hunter, 1999) recognized the negative impact of such isolation and spoke to the buffering effect of connection with those whose experiences closely match their own; however, this was often difficult to find.

Threatened female identity/femininity was recognized by 42% of studies (*n* = 5). The physical and psychological changes experienced in menopause, alongside the stereotypes of the menopausal woman, threatened the women's identity associated with femininity, womanhood and/or motherhood. The experience of menopause was seen as not aligning with the societal ideals around womanhood and femininity (i.e., young, attractive, fertile), underscoring the fragility of such ideals (Singer & Hunter, 1999). Amenorrhea and infertility were experienced as substantial losses for those with or without children. For many women, fertility was strongly connected with womanhood and femininity, with such changes leading them to not feel like a ‘*proper*’ or ‘*real*’ woman (Singer, 2012; Singer & Hunter, 1999). Singer and Hunter (1999, p. 70) reported on the experience of this in their study, ‘*More generally it was felt, although to differing degrees, that a “complete” woman is one who is at least potentially fertile*.’ For some women loss of fertility challenged identities associated with motherhood. Despite not specifically planning to have children, projected identity of motherhood was impacted by the lack of choice and/or no longer being fertile. One woman in Boughton's (2002, p. 429) study spoke to the shift in the way she experienced her identity in terms of motherhood:

‘The sad thing is that I can't have children any more…It's all that sort of image of you as the earth mother, you know, bearing fruit and all those images that we're supposed to symbolize—that's no longer there. So, you need to find another sort of identity—not being a mother and that's something I find difficult to deal with.’

Challenged sexual identity was reported on in 42% of studies (*n* = 5). Sexual identity often felt challenged due to shifts in sexual relationships due to physical and emotional changes, social norms pertaining to sex and/or the perceptions of the ‘menopausal women’. Experiences of menopause such as hot flushes, vaginal dryness and decreased libido, often felt misaligned with sex and intimacy, making it difficult to think of themselves as a ‘*sexual being*’ (Johnston‐Ataata et al., 2020). Generally, responses from themselves and partners influenced this experience (Johnston‐Ataata et al., 2020; Golezar et al., 2020). Acceptance often aided transition to a reimagined sexual identity (Johnston‐Ataata et al., 2020), whereas more traditional views around sexuality and relationships, and negative judgements from partner's often led individuals to dishonour their bodies and feel disconnected from their sexual identity. Social constructions of the menopausal individual as less feminine, less desirable and asexual or less sexual felt threatening to some of the women's sexuality. Internalization of such stereotypes impacted how the women experienced or expressed their sexuality, often leading to feelings of embarrassment and shame around their sexual identities (Singer & Hunter, 1999).

### Emotional self‐concept

Impacts to emotional self‐concept made up 30% of all category references. Disrupted life trajectory or plans were described by 58% of studies (*n* = 7), indicating how the experiences of POI and EM were often perceived to interfere with imagined life trajectories and plans. Disruptions varied in terms of scale. For some, their daily life was immediately impacted, with symptoms making things they usually were able to do, such as going to work, parenting and managing the house, unmanageable (Boughton, 2002). Often the disruptions were associated with long‐term ongoing losses, associated with considerable grief—grieving the person they “should” have been (Golezar et al., 2020, p. 6 ‘women experienced grief for quote a long time after being diagnosed with the disease… they were concerned about the complications of the disease… and couldn't easily talk about it’).

Such experience is described by one of the younger participants in Singer's (2012, p. 104) study, ‘It's hard to know how I would have been without the menopause. I've grown up…feeling life is over before it started’. With this, some of the women experience a lack of control over their lives, with choice around certain decisions being taken away (Singer, 2012; Singer & Hunter, 1999). The disruption to family plans and having children was especially impactful, as expressed by a woman in Singer & Hunter (1999, p. 70), ‘It's a bit of a bereavement feeling. I realised I hadn't dealt with the feeling of loss. I dealt with the practical side of things, but emotionally I was crippling myself’. Such bereavement was associated with the realization that something can never or no longer be. In response to such disruptions, especially as time went on, women attempted to reimage their future roles or plans (Johnston‐Ataata et al., 2020; Singer & Hunter, 1999).

Seven studies (58%) reported on erosion of self‐esteem and self‐confidence, detailing the negative impact of the changes and losses experienced with POI and EM. Self‐esteem was shown to be more negatively impacted for those with POI and EM than those experiencing normal menopause (Ryu et al., 2022), or relatively low compared to age‐related norms (Singer, 2012). Impacts to self‐esteem and self‐confidence were often associated with the feeling of being abnormal or lacking something compared to others (Golezar et al., 2020; Johnston‐Ataata et al., 2020; Singer, 2012; Singer & Hunter, 1999). Often women would blame themselves or question whether such experiences reflected personal failures (Singer, 2012; Singer & Hunter, 1999). For some women, the sense of failure lessened over time (Singer, 2012).

### The role of contextual factors

As identified in Table 2, the studies, to varying degrees, suggest that the extent of impact to self‐concept and related constructs is influenced by various interconnected factors relating to the women's context, both societally and individually, such as identification with societal norms, stereotypes, stigma, age, cause, symptoms, children, relationships, psychological support and time since diagnosis. Traditional norms around reproduction, femininity and/or age, and identification with such norms, were often associated with a greater sense of loss and impact to self‐concept and related constructs such as self‐confidence, body‐image, self‐ and social‐identity (Golezar et al., 2020; Johnston‐Ataata et al., 2020; Singer & Hunter, 1999). The attachment to such norms from family, partners and friends also played a role. The sample in Johnston‐Ataata et al.'s (2020) study showed variation in experiences. Those whose feminine identity was not strongly tied to social norms or who questioned such norms experienced a smoother transition to their menopause self, while those more rigidly attached to norms often experienced such changes in terms of loss. Golezar et al. (2020) indicated that impacts for Iranian women were predominantly negative as a key part of the Iranian women's cultural identity was tied to having children and filling a maternal role.

Many studies reported on the impact of the negative stereotypes and stigma associated with menopause, often influencing a sense of isolation from peers and the self (Boughton, 2002; Boughton & Halliday, 2008; Golezar et al., 2020; Johnston‐Ataata et al., 2020; Singer, 2012; Singer & Hunter, 1999). Boughton and Halliday (2008) described how stigma and stereotypes around age and image of the menopausal woman contributed to participants' altered self‐perceptions, with feelings of abnormality and distance from peers. Johnston‐Ataata et al. (2020) reported that for some women in their study, sharing their experiences helped them to challenge stereotypes and accept their menopausal selves.

Several studies indicated that age has an impact on women's experiences, with menopause at a younger age often being experienced as a greater disruption to life‐stage or trajectory, fostering increased feelings of separation from peers and themselves (Boughton, 2002; Boughton & Halliday, 2008; Johnston‐Ataata et al., 2020; Liao et al., 2000; Ryu et al., 2022; Singer, 2012). Symptoms which were more severe or difficult to manage were associated with increased negative impacts on body‐image and self‐confidence (Johnston‐Ataata et al., 2020). Experiences of menopausal symptoms, for some, served as indicators of the potential damage occurring to the body and/or reinforced a sense of confusion or disconnect towards their body (Boughton, 2002; Boughton & Halliday, 2008). Women across many studies described the impact of not being able to have children, or additional children (Boughton, 2002; Boughton & Halliday, 2008; Golezar et al., 2020; Johnston‐Ataata et al., 2020; Liao et al., 2000; Singer, 2012; Singer & Hunter, 1999). Boughton (2002) suggested that the female identity was threatened for those with or without children, whereas identity connected to motherhood was primarily impacted for those without children.

## DISCUSSION

The current scoping review identified impacts across core areas of self‐concept (i.e., physical, social, emotional). The specific impacts were grouped into eight categories (e.g., body out of synchrony, disrupted life trajectory or plans, threatened female identity/femininity), with categories relevant to more than one aspect of self‐concept, reflecting the interconnected nature of the individual domains of self‐concept and the experiences of POI and EM (Hunter & Edozien, 2017; Mercer, 2012; Shavelson et al., 1976). Implications for physical self‐concept were most frequently described across the reviewed studies, suggesting that POI and EM significantly shape individuals embodied experience of these conditions. This aligns with the significant physical changes associated with POI and EM (Faubion et al., 2015; Hammond & Marczak, 2025; Li et al., 2020), which are often experienced as disruptive and distressing (Hammond & Marczak, 2025; Singer, 2019). Existing findings have noted a link between menopause and negative perceptions of both the body and self, with such perceptions often associated with menopausal symptoms themselves and the cultural and societal meanings attached to menopause (Vincent et al., 2023; Walter, 2000).

The prevailing stereotype of menopause or the ‘menopausal woman’ is often internalized by those with POI, EM and naturally occurring menopause, as efforts to make sense of their experiences are shaped by negative imagery of ageing centred around loss of youth, fertility, physical attractiveness and sexual desire (De Boer & Halsema, 2024; Vincent et al., 2025; Walter, 2000). Studies suggest that the impact of such reckoning on body image and self‐perception is often substantial due to the sociocultural expectations placed on women, and those assumed female at birth, to conform to an ‘ideal’ image (Vincent et al., 2025; Walter, 2000). Notably, other experiences relating to reproduction such as puberty, pregnancy, postpartum and naturally occurring menopause reflect periods of vulnerability to body image concerns (Spinoni et al., 2023; Vincent et al., 2025). However, POI and EM encompass unique experiences such as age of onset and timing of diagnosis, which also influence body image, self‐perception and the broader domain of physical self‐concept (Boughton, 2002; Singer, 2012; Singer & Hunter, 1999).

The participants in the reviewed studies often indicated that their physical body was out of synchrony with their age and/or was problematic and not to be trusted. While naturally occurring menopause is associated with negative depictions of ageing, such fears appear to be exacerbated for those with POI and EM due to the lack of comparability with their same age peers (Hoga et al., 2015; Walter, 2000). Specifically, an increased sense of premature ageing was influenced by the polarity between a young adult identity and the perception of a rapidly changing and ageing body due to POI and EM. This experience is comparable to findings focused on individuals with chronic illness where they describe a sense of feeling older than their chronological age, with the associated physical experiences contributing to the embodiment of premature ageing (Giddings et al., 2007; Weeks et al., 2003). Similarly, the sense of distrust towards the body reflected in the current review is also consistent with distrust towards the body found in individuals living with chronic illness (Giddings et al., 2007; Hajdarevic et al., 2025). Such similarities between the experiences of POI and EM and chronic illness may reflect the differing perception of POI and EM versus naturally occurring menopause.

While historically the medicalization of menopause has framed menopause around the breakdown of the reproductive system alongside terms such as ‘failure’ and ‘deficiency’ such conceptualization is gradually shifting (Pasquali, 1999; Wood et al., 2025). Increasingly, menopause is recognized as a normal part of life, with a slow but growing understanding and acknowledgement that those with menopausal symptoms should not be left to struggle, but rather have access to medical intervention, such as HRT, to support such transition (Davis et al., 2023; Wood et al., 2025). However, despite this gradual shift, POI and EM continue to be framed as abnormal and centre around loss and risk (Hammond & Marczak, 2025; Lawlor et al., 2002; Pasquali, 1999; Singer, 2012). For instance, in the 12 reviewed studies, those with POI and EM report viewing their bodies as sites of sickness due to their experiences not fitting the expected timeline of natural menopause (Boughton & Halliday, 2008; Singer & Hunter, 1999).

The 12 reviewed studies also indicated broad ranging impacts to the social domain of self‐concept, often noting a sense of ‘otherness’ or ‘lacking’. Predominantly this was a result of the experiences of POI and EM not aligning with cultural and societal ideals around age, femininity, womanhood and sexuality. Such experiences, as recognized by Johnston‐Ataata et al. (2020), align with the notion of biographical disruption, which was first described by Bury (1982) in relation to the experience of chronic illness. Biographical disruption describes how illness can cause significant disruption in everyday life as well as the imagined or projected life trajectories through which the individual understands themselves and their life through (Bury, 1982). Accordingly, many of the participants in the reviewed studies experienced a significant rupture in their imagined biographies, influencing how they view and understand themselves in relation to others. Certainly, within other diagnoses such as breast or gynaecological cancers and endometriosis, women's experiences are shaped by embedded societal norms equating femininity with reproductive capacity, bodily control and expected life trajectories (Sun et al., 2018). When illness disrupts areas such as fertility, sexual function or expectations of motherhood, these culturally prescribed ideals may be internalized, leading to feelings of shame, inadequacy and ‘failure’ (Brania et al., 2026).

The sense of being different extended beyond the physical body itself for the women in the reviewed studies, with a sense of inferiority to their peers felt due to the emotional and physical changes of POI and EM. Such interpretations were often influenced by norms around age or time of life and perpetuated by confusion and a sense of not necessarily ‘belonging’ to their age‐matched social groupings (Boughton, 2002; Boughton & Halliday, 2008; Golezar et al., 2020; Johnston‐Ataata et al., 2020; Singer, 2012). Fertility‐based literature citing concerns noted by women experiencing infertility is like that of the current review finding, whereby seeing their peers' forming families or talking about their future with children maintained a sense of inferiority, being different or not belonging (Assaysh‐Öberg et al., 2023; Lindsey & Driskill, 2013). Notably, when women were able to connect with others experiencing POI or EM, and see shared experiences, it often acted as a buffer to feelings of otherness (Johnston‐Ataata et al., 2020; Singer & Hunter, 1999). Some participants spoke to internalizing sexual desire stereotypes and others reported that the fertility loss or insecurity impacted their identity as fertility is often tied to sense of womanhood and motherhood. Such identity disruption or alteration in projected life trajectories is well documented across experiences of infertility (Alamin et al., 2020; Greil et al., 2010).

Impacts to the emotional domain of self‐concept were noted across the 12 reviewed studies, with alterations within this domain including the emotional consequences of POI and EM. Bury's (1982) biographical disruption provides context to the reported outcomes, whereby disruption to the projected life trajectory leads to significant shifts in how one perceives their emotional self – leading to emotional experiences of grief and loss – negatively impacting self‐esteem and broader self‐concept. As indicated previously in relation to the physical and social consequences of POI and EM and shared similarities with those with chronic illness, the personal and emotional impacts also share similarities in the experiences of unexpected losses or disruptions and sense of distance from peers. Loss and grief associated with the view of who they ‘could have’ or ‘should have been’ was a common experience (Culley et al., 2013; Wilkins, 2001; Young et al., 2015). This was especially mirrored in studies around endometriosis, where plans around having children can be similarly disrupted (Culley et al., 2013; Young et al., 2015).

Existing findings relating to both menopause and chronic illness reference the emotions that individuals report as they attempt to understand and make meaning of menopause or illness‐related experiences, often recounting high distress in the initial phase and moving towards a greater level of acceptance as time goes on (de Salis et al., 2017; Morrison et al., 2013; Wilkins, 2001; Yang et al., 2016). While the reviewed studies suggested that impacts to self‐esteem and self‐confidence were experienced to a greater degree for women with POI and EM compared to naturally occurring menopause (Ryu et al., 2022), shifts from emotional distress towards greater acceptance were noted. During the initial stages, some women reportedly blamed themselves or felt that their diagnosis reflected a personal failure but indicated that negative views towards the self gradually decreased with time (Singer, 2012; Singer & Hunter, 1999). The shift from distress and a negative view of self towards one of acceptance aligns with the process of biographical repair, which has been recognized in the literature as a process following biographical disruption where there is an attempt to reconstruct or reimage identity and/or the self along with physical changes (Bury, 1982; Locock et al., 2009).

Evident in the reviewed studies was the importance of considering the role that other individual or contextual factors have on the impact on self‐concept in POI and EM, alongside the cause. All studies, to varying degrees, indicated that the extent to which areas of self‐concept are influenced by POI and EM is associated with various interconnected factors relating to social and personal context. Among the reviewed studies, Johnston‐Ataata et al. (2020) emphasized the need to account for individual or contextual factors, with their findings suggesting that the cause of POI and EM was not a stand‐alone influence but rather intertwined with various factors. Such findings emphasize the importance of considering potential alterations to self‐concept associated with POI and EM through a biopsychosocial lens (Hunter & Edozien, 2017).

## LIMITATIONS

The current scoping review was limited by a few factors. Firstly, while the review aimed to provide data on the impacts to self‐concept experienced in POI and EM, much of the data focused predominantly on POI. As such, this limits the ability to differentiate between the experiences of each diagnosis separately, with less support for conclusions drawn about EM compared to POI. This may be due to various factors, one being that POI includes a bigger age range than EM, potentially making it easier to source participants. Another reason is connected to a second limitation. Due to variation in menopause terminology over time and contexts, it was often difficult to determine whether included participants met POI and EM diagnoses. For instance, some authors referred to ‘premature menopause’ to describe medically induced menopause, reflecting menopause that was not naturally occurring, whereas other authors directly referred to POI. Furthermore, while some studies included participants who may have met criteria for POI and EM, results were not differentiated between other participants who experienced menopause at 50 years or older. In addition to the lack of diagnostic differentiation reported in study samples, some studies reported limited inclusion or exclusion criteria or reported on participant age at the time of study but not the age at menopause or diagnosis. More detailed inclusion and exclusion criteria, consistent terminology and definitions, and diagnostic differentiation within study samples may help in mitigating these limitations in future research.

Lastly, the current scoping review was limited by the lack of sample diversity in the included studies, especially in terms of ethnicity and cultural background and sexuality, with most studies including participants who were from white western backgrounds, heterosexual and cisgender. This limits the ability to generalize the conclusions found in the current review beyond cisgender heteronormative populations. However, the current authors note that there was some variation in the cultural experiences reflected in the reviewed studies, underscoring the importance of such diversity, with such contextual factors having an impact on experiences of POI and EM and self‐concept.

## FUTURE DIRECTIONS

As research into POI and EM remains limited, several areas warrant further attention. Future studies could incorporate measures that capture the multidimensional nature of self‐concept. The Tennessee Self‐Concept Scale (TSCS‐2; Fitts & Warren, 1996) may be particularly appropriate for adults with POI or EM, as it assesses self‐concept across interrelated domains (e.g., physical, personal and social self). Women with POI and EM are known to experience overlapping disruptions in body image, identity and psychological wellbeing. As such, the TSCS‐2 is well aligned with the complex, multidimensional nature of self‐concept in this group and may support more targeted clinical assessment and intervention. The diagnostic experience and ongoing care also require further investigation, as many studies highlight negative diagnostic encounters and limited psychological support. Improving these aspects of health care should be a key focus. Additionally, given evidence that connections with other women with shared experiences can reduce feelings of isolation and otherness, future research could explore the development and efficacy of peer‐based or group interventions. Such approaches may help address impacts on self‐concept and reduce internalized shame in POI and EM populations.

## CONCLUSION

In summary, this scoping review highlights the significant impacts that POI and EM can have on multiple domains of self‐concept—physical, social and emotional. Individuals with POI and EM often experience similar difficulties, which reflect the significant disruption that these diagnoses may have on projected life trajectories. However, the severity of self‐concept impacts appears to depend on various contextual factors including the cause of menopause, symptoms and identification with societal norms. While changes in terminology and research approaches potentially limit the current findings to cisgender heteronormative individuals, there is further need for research that includes greater sample diversity. Further investigation of the availability of person‐centred health care, both physical and psychological and the patient‐health care practitioner relationship is recommended to address the identified self‐concept implications of POI and EM.

## AUTHOR CONTRIBUTIONS

**Lisa M. Seberry:** Conceptualization; methodology; data curation; formal analysis; investigation; writing – original draft; writing – review and editing; software. **Subhadra Evans:** Writing – original draft; writing – review and editing. **Antonina Mikocka‐Walus:** Writing – original draft; writing – review and editing. **Leesa M. Van Niekerk:** Conceptualization; methodology; software; data curation; investigation; formal analysis; supervision; writing – original draft; writing – review and editing.

## FUNDING INFORMATION

This research did not receive any specific grant from funding agencies in the public, commercial or non‐profit sectors.

## CONFLICT OF INTEREST STATEMENT

The authors declare that they have no known competing financial interests or personal relationships that could have appeared to influence the work reported in this paper.

## Data Availability

Data sharing not applicable to this article as no datasets were generated or analysed during the current study.

## References

[bjhp70104-bib-0001] Alamin, S. , Allahyari, T. , Ghorbani, B. , Sadeghitabar, A. , & Karami, M. T. (2020). Failure in identity building as the main challenge of infertility: A qualitative study. Journal of Reproduction & Infertility, 21(1), 49. https://pmc.ncbi.nlm.nih.gov/articles/PMC7048687/ 32175265 PMC7048687

[bjhp70104-bib-0002] Assaysh‐Öberg, S. , Borneskog, C. , & Ternström, E. (2023). Women's experience of infertility & treatment – A silent grief and failed care and support. Sexual & Reproductive Healthcare, 37, 100879. 10.1016/j.srhc.2023.100879 37356208

[bjhp70104-bib-0064] Bailey, J. A., 2nd . (2003). Self‐image, self‐concept, and self‐identity revisited. Journal of the National Medical Association, 95(5), 383. 12793794 PMC2594523

[bjhp70104-bib-0004] Boughton, M. A. (2002). Premature menopause: multiple disruptions between the woman's biological body experience and her lived body. Journal of Advanced Nursing, 37(5), 423–430. 10.1046/j.1365-2648.2002.02114.x 11843980

[bjhp70104-bib-0003] Boughton, M. , & Halliday, L. (2008). A challenge to the menopause stereotype: Young Australian women's reflections of ‘being diagnosed’ as menopausal. Health & Social Care in the Community, 16(6), 565–572. 10.1111/j.1365-2524.2008.00777.x 18371169

[bjhp70104-bib-0005] Brania, N. , Adler, H. , Richardson, E. , Ng, C. , O'Hara, R. , Pirotta, S. , & Van Niekerk, L. (2026). Debunking the “mystical condition” of endometriosis: What people living with endometriosis want you to truly understand. Women's Reproductive Health, 13, 1–17. 10.1080/23293691.2025.2525429

[bjhp70104-bib-0006] Bury, M. (1982). Chronic illness as biographical disruption. Sociology of Health & Illness, 4(2), 167–182. 10.1111/1467-9566.ep11339939 10260456

[bjhp70104-bib-0007] Charmaz, K. (2002). The self as habit: The reconstruction of self in chronic illness. OTJR: Occupation, Participation and Health, 22(1), 31S–41S. 10.1177/15394492020220S105

[bjhp70104-bib-0008] Culley, L. , Law, C. , Hudson, N. , Denny, E. , Mitchell, H. , Baumgarten, M. , & Raine‐Fenning, N. (2013). The social and psychological impact of endometriosis on women's lives: A critical narrative review. Human Reproduction Update, 19(6), 625–639. 10.1093/humupd/dmt027 23884896

[bjhp70104-bib-0009] Davis, S. R. , Pinkerton, J. , Santoro, N. , & Simoncini, T. (2023). Menopause – Biology, consequences, supportive care, and therapeutic options. Cell, 186(19), 4038–4058. 10.1016/j.cell.2023.08.016 37678251

[bjhp70104-bib-0010] De Boer, M. , & Halsema, A. (2024). Mimicking myths of menopause. A critical phenomenological perspective on ageing and femininity in fiction TV shows. *Philosophy & Social Criticism*. 1–19. 10.1177/01914537241232586

[bjhp70104-bib-0011] de Salis, I. , Owen‐Smith, A. , Donovan, J. L. , & Lawlor, D. A. (2017). Experiencing menopause in the UK: The interrelated narratives of normality, distress, and transformation. Journal of Women & Aging, 30(6), 520–540. 10.1080/08952841.2018.1396783 29095126 PMC6191885

[bjhp70104-bib-0012] Deeks, A. , Gibson‐Helm, M. , Teede, H. , & Vincent, A. (2011). Premature menopause: A comprehensive understanding of psychosocial aspects. Climacteric, 14(5), 565–572. 10.3109/13697137.2011.566390 21854296

[bjhp70104-bib-0013] Faubion, S. S. , Kuhle, C. L. , Shuster, L. T. , & Rocca, W. A. (2015). Long‐term health consequences of premature or early menopause and considerations for management. Climacteric, 18(4), 483–491. 10.3109/13697137.2015.1020484 25845383 PMC4581591

[bjhp70104-bib-0014] Fitts, W. H. , & Warren, W. L. (1996). Tennessee Self‐Concept Scale: Second Edition (TSCS‐2). Western Psychological Services.

[bjhp70104-bib-0015] Giddings, L. S. , Roy, D. E. , & Predeger, E. (2007). Women's experience of ageing with a chronic condition. Journal of Advanced Nursing, 58(6), 557–565. 10.1111/j.1365-2648.2007.04243.x 17484749

[bjhp70104-bib-0016] Golezar, S. , Keshavarz, Z. , Ramezani Tehrani, F. , & Ebadi, A. (2020). An exploration of factors affecting the quality of life of women with primary ovarian insufficiency: A qualitative study. BMC Women's Health, 20, 1–9. 10.1186/s12905-020-01029-y 32758224 PMC7405332

[bjhp70104-bib-0017] Gosset, A. , Claeys, J. M. , Huyghe, E. , & Tremollieres, F. (2023). Sexual function and quality of life in women with idiopathic premature ovarian insufficiency. The Journal of Sexual Medicine, 20(5), 626–632. 10.1093/jsxmed/qdad006 36881744

[bjhp70104-bib-0018] Greil, A. L. , Slauson‐Blevins, K. , & McQuillan, J. (2010). The experience of infertility: A review of recent literature. Sociology of Health & Illness, 32(1), 140–162. 10.1111/j.1467-9566.2009.01213.x 20003036 PMC3383794

[bjhp70104-bib-0019] Guerrieri, G. M. , Martinez, P. E. , Klug, S. P. , Haq, N. A. , Vanderhoof, V. H. , Koziol, D. E. , Popat, V. B. , Kalantaridou, S. N. , Calis, K. A. , & Rubinow, D. R. (2014). Effects of physiologic testosterone therapy on quality of life, self‐esteem, and mood in women with primary ovarian insufficiency. Menopause, 21(9), 952–961. 10.1097/GME.0000000000000195 24473536 PMC4112175

[bjhp70104-bib-0020] Hajdarevic, S. , Norberg, A. , Lundman, B. , & Hörnsten, Å. (2025). Becoming whole again – Caring for the self in chronic illness – A narrative review of qualitative empirical studies. Journal of Clinical Nursing, 34(3), 754–771. 10.1111/jocn.17332 38886987 PMC11808418

[bjhp70104-bib-0021] Hammond, J. , & Marczak, M. (2025). Women's experiences of premature ovarian insufficiency: A thematic synthesis. Psychology & Health, 40(2), 192–216. 10.1080/08870446.2023.2192738 36971566

[bjhp70104-bib-0022] Hamoda, H. , & Sharma, A. (2024). Premature ovarian insufficiency, early menopause, and induced menopause. Best Practice & Research Clinical Endocrinology & Metabolism, 38(1), 101823. 10.1016/j.beem.2023.101823 37802711

[bjhp70104-bib-0070] Harter, S. (1999). The construction of the self: A developmental perspective. Guilford press.

[bjhp70104-bib-0023] Hoga, L. , Rodolpho, J. , Gonçalves, B. , & Quirino, B. (2015). Women's experience of menopause: A systematic review of qualitative evidence. JBI Evidence Synthesis, 13(8), 250–337. 10.11124/jbisrir-2015-1948 26455946

[bjhp70104-bib-0025] Hunter, M. S. , & Edozien, L. C. (2017). Special issue on biopsychosocial perspectives on the menopause. Journal of Psychosomatic Obstetrics & Gynecology, 38(3), 159–160. 10.1080/0167482X.2017.1358135 28745148

[bjhp70104-bib-0026] Johnston‐Ataata, K. , Flore, J. , Kokanović, R. , Hickey, M. , Teede, H. , Boyle, J. A. , & Vincent, A. (2020). ‘My relationships have changed because I’ve changed’: biographical disruption, personal relationships and the formation of an early menopausal subjectivity. Sociology of Health & Illness, 42(7), 1516–1531. 10.1111/1467-9566.13143 32584443

[bjhp70104-bib-0027] Lawlor, D. A. , Adamson, J. , & Ebrahim, S. (2002). Lay perceptions of a ‘natural’ menopause. Cross sectional study of the British Women's heart and health study. BJOG: An International Journal of Obstetrics & Gynaecology, 109(12), 1398–1400. 10.1046/j.1471-0528.2002.02046.x 12504978

[bjhp70104-bib-0028] Li, X. , Li, P. , Liu, Y. , Yang, H. , He, L. , Fang, Y. , Liu, J. , Liu, B. , & Chaplin, J. (2020). Health‐related quality‐of‐life among patients with premature ovarian insufficiency: A systematic review and meta‐analysis. Quality of Life Research, 29, 19–36. 10.1007/s11136-019-02326-2 31620985 PMC6962283

[bjhp70104-bib-0029] Liao, K. L. , Wood, N. , & Conway, G. (2000). Premature menopause and psychological well‐being. Journal of Psychosomatic Obstetrics & Gynecology, 21(3), 167–174. 10.3109/01674820009075624 11076338

[bjhp70104-bib-0030] Lindsey, B. , & Driskill, C. (2013). The psychology of infertility. International Journal of Childbirth Education, 28(3), 41–47.

[bjhp70104-bib-0031] Locock, L. , Ziebland, S. , & Dumelow, C. (2009). Biographical disruption, abruption and repair in the context of motor neurone disease. Sociology of Health & Illness, 31(7), 1043–1058. 10.1111/j.1467-9566.2009.01176.x 19659736

[bjhp70104-bib-0032] Loxton, D. , Tooth, L. , Barnes, I. , Byrnes, E. , Cavenagh, D. , Chung, H.‐F. , Egan, N. , Forder, P. , Harris, M. , & Hockey, R. (2021). Reproductive health: contraception, conception and change of life‐findings from the Australian Longitudinal Study on Women's Health. Women's Health Australia. https://espace.library.uq.edu.au/view/UQ:35af26d

[bjhp70104-bib-0033] Mazalin, K. , Evans, S. , & Van Niekerk, L. (2024). A template thematic analysis of self‐concept and self‐compassion in people living with endometriosis: Analysis of qualitative survey responses. Journal of Advanced Nursing, 81, 4216–4227. 10.1111/jan.16645 39618124 PMC12159396

[bjhp70104-bib-0034] McDonald, I. R. , Welt, C. K. , & Dwyer, A. A. (2022). Health‐related quality of life in women with primary ovarian insufficiency: A scoping review of the literature and implications for targeted interventions. Human Reproduction, 37(12), 2817–2830. 10.1093/humrep/deac200 36102839 PMC9989734

[bjhp70104-bib-0035] Mercer, S. (2012). Self‐concept: Situating the self. In Psychology for Language Learning: Insights From Research, Theory and Practice (pp. 10–25). Springer. 10.1057/9781137032829_2

[bjhp70104-bib-0036] Moadel, A. B. , Ostroff, J. S. , Lesko, L. M. , & Bajorunas, D. R. (1995). Psychosexual adjustment among women receiving hormone replacement therapy for premature menopause following cancer treatment. Psycho‐Oncology, 4(4), 273–282. 10.1002/pon.2960040404

[bjhp70104-bib-0037] Morrison, L. A. , Brown, D. E. , Sievert, L. L. , Reza, A. , Rahberg, N. , Mills, P. , & Goodloe, A. (2013). Voices from the Hilo Women's health study: Talking story about menopause. Health Care for Women International, 35(5), 529–548. 10.1080/07399332.2013.829067 24134306 PMC3925469

[bjhp70104-bib-0038] Moukhah, S. , Ghorbani, B. , Behboodi‐Moghadam, Z. , & Zafardoust, S. (2021). Perceptions and experiences of women with premature ovarian insufficiency about sexual health and reproductive health. BMC Women's Health, 21(1), 54. 10.1186/s12905-021-01197-5 33557799 PMC7869211

[bjhp70104-bib-0039] Orshan, S. A. , Furniss, K. K. , Forst, C. , & Santoro, N. (2001). The lived experience of premature ovarian failure. Journal of Obstetric, Gynecologic, and Neonatal Nursing, 30(2), 202–208. 10.1111/j.1552-6909.2001.tb01536.x 11308110

[bjhp70104-bib-0040] Ossewaarde, M. E. , Bots, M. L. , Verbeek, A. L. , Peeters, P. H. , van der Graaf, Y. , Grobbee, D. E. , & van der Schouw, Y. T. (2005). Age at menopause, cause‐specific mortality and total life expectancy. Epidemiology, 16(4), 556–562. 10.1097/01.ede.0000165392.35273.d4 15951675

[bjhp70104-bib-0065] Page, M. J. , McKenzie, J. E. , Bossuyt, P. M. , Boutron, I. , Hoffmann, T. C. , Mulrow, C. D. , Shamseer, L. , Tetzlaff, J. M. , Akl, E. A. , & Brennan, S. E. (2021). The PRISMA 2020 statement: An updated guideline for reporting systematic reviews. BMJ, 372. 10.1136/bmj.n71 PMC8005924 33782057

[bjhp70104-bib-0041] Pal, L. , & Santoro, N. (2002). Premature ovarian failure (POF): Discordance between somatic and reproductive aging. Ageing Research Reviews, 1(3), 413–423. 10.1016/S1568-1637(02)00009-0 12067595

[bjhp70104-bib-0042] Pasquali, E. A. (1999). The impact of premature menopause on women's experience of self. Journal of Holistic Nursing, 17(4), 346–364. 10.1177/089801019901700404 10818847

[bjhp70104-bib-0043] Peters, M. D. J. , Godfrey, C. , McInerney, P. , Munn, Z. , Tricco, A. C. , & Khalil, H. (2020). Scoping reviews. In E. Aromataris , C. Lockwood , K. Porritt , B. Pilla , & Z. Jordan (Eds.), JBI Manual for Evidence Synthesis (Vol. 2024). JBI. 10.46658/JBIMES-24-09

[bjhp70104-bib-0044] Ryu, K.‐J. , Park, H. , Jeong, Y. , Nam, S. , Jeong, H. G. , & Kim, T. (2022). Age at menopause and suicidal ideation in menopausal women: A study of Korea National Health and nutrition examination survey data. Journal of Korean Medical Science, 37(45), e330. 10.3346/jkms.2022.37.e330 36413799 PMC9678656

[bjhp70104-bib-0045] Schmidt, P. J. , Cardoso, G. M. , Ross, J. L. , Haq, N. , Rubinow, D. R. , & Bondy, C. A. (2006). Shyness, social anxiety, and impaired self‐esteem in turner syndrome and premature ovarian failure. JAMA, 295(12), 1373–1378. 10.1001/jama.295.12.1374 16551707

[bjhp70104-bib-0046] Shavelson, R. J. , Hubner, J. J. , & Stanton, G. C. (1976). Self‐concept: Validation of construct interpretations. Review of Educational Research, 46(3), 407–441. 10.3102/00346543046003407

[bjhp70104-bib-0047] Shuster, L. T. , Rhodes, D. J. , Gostout, B. S. , Grossardt, B. R. , & Rocca, W. A. (2010). Premature menopause or early menopause: Long‐term health consequences. Maturitas, 65(2), 161–166. 10.1016/j.maturitas.2009.08.003 19733988 PMC2815011

[bjhp70104-bib-0048] Singer, D. (2012). ‘It's not supposed to be this way’: Psychological aspects of a premature menopause. Counselling and Psychotherapy Research, 12(2), 100–108. 10.1080/14733145.2011.648202

[bjhp70104-bib-0049] Singer, D. (2019). Managing the psychological sequelae of POI. Post Reproductive Health, 25(3), 150–155. 10.1177/2053369119874640 31630612

[bjhp70104-bib-0050] Singer, D. , & Hunter, M. (1999). The experience of premature menopause: A thematic discourse analysis. Journal of Reproductive and Infant Psychology, 17(1), 63–81. 10.1080/02646839908404585

[bjhp70104-bib-0051] Singer, D. , Mann, E. , Hunter, M. , Pitkin, J. , & Panay, N. (2011). The silent grief: Psychosocial aspects of premature ovarian failure. Climacteric, 14(4), 428–437. 10.3109/13697137.2011.571320 21762006

[bjhp70104-bib-0052] Spinoni, M. , Singh Solorzano, C. , & Grano, C. (2023). A prospective study on body image disturbances during pregnancy and postpartum: The role of cognitive reappraisal. Frontiers in Psychology, 14, 1200819. 10.3389/fpsyg.2023.1200819 37621944 PMC10444978

[bjhp70104-bib-0053] Sun, L. , Ang, E. , Ang, W. H. D. , & Lopez, V. (2018). Losing the breast: A meta‐synthesis of the impact in women breast cancer survivors. Psycho‐Oncology, 27(2), 376–385. 10.1002/pon.4460 28544436

[bjhp70104-bib-0054] Veritas Health Innovation . (2021). Covidence Systematic Review Software. Veritas Health Innovation. https://www.covidence.org/

[bjhp70104-bib-0055] Vincent, C. , Bodnaruc, A. M. , Prud'homme, D. , Olson, V. , & Giroux, I. (2023). Associations between menopause and body image: A systematic review. Women's Health, 19, 17455057231209536. 10.1177/17455057231209536 PMC10666711 37994043

[bjhp70104-bib-0056] Vincent, C. , Ménard, A. , & Giroux, I. (2025). Cultural determinants of body image: What about the menopausal transition? Health, 13(1), 76. 10.3390/healthcare13010076 PMC11720564 39791683

[bjhp70104-bib-0057] Walter, C. A. (2000). The psychosocial meaning of menopause: women's experiences. Journal of Women & Aging, 12(3–4), 117–131. 10.1300/J074v12n03_08 11151348

[bjhp70104-bib-0058] Webber, L. , Davies, M. , Anderson, R. , Bartlett, J. , Braat, D. , Cartwright, B. , Cifkova, R. , de Muinck Keizer‐Schrama, S. , & Hogervorst, E. (2016). ESHRE guideline: Management of women with premature ovarian insufficiency. Human Reproduction, 31(5), 926–937. 10.1093/humrep/dew027 27008889

[bjhp70104-bib-0059] Weeks, A. , McAvoy, B. , Peterson, C. , Furler, J. , Walker, C. , Swerissen, H. , & Belfrage, J. (2003). Negotiating ownership of chronic illness: An appropriate role for health professionals in chronic illness self‐management programs. Australian Journal of Primary Health, 9(3), 25–33. 10.1071/PY03020

[bjhp70104-bib-0060] Wilkins, S. (2001). Aging, chronic illness and self‐concept: A study of women with osteoporosis. Journal of Women & Aging, 13(1), 73–92. 10.1300/J074v13n01_06 11217187

[bjhp70104-bib-0061] Wood, K. , McCarthy, S. , Pitt, H. , Randle, M. , & Thomas, S. L. (2025). Women's experiences and expectations during the menopause transition: A systematic qualitative narrative review. Health Promotion International, 40(1), daaf005. 10.1093/heapro/daaf005 40036278 PMC11878557

[bjhp70104-bib-0062] Yang, C. F. , Kenney, N. J. , Chang, T. C. , & Chang, S. R. (2016). Sex life and role identity in Taiwanese women during menopause: A qualitative study. Journal of Advanced Nursing, 72(4), 770–781. 10.1111/jan.12866 26708447

[bjhp70104-bib-0063] Young, K. , Fisher, J. , & Kirkman, M. (2015). Women's experiences of endometriosis: A systematic review and synthesis of qualitative research. The Journal of Family Planning and Reproductive Health Care, 41(3), 225–234. 25183531 10.1136/jfprhc-2013-100853

